# Affective Touch Reduces Electrically Induced Itch Experience

**DOI:** 10.3389/fmed.2021.628020

**Published:** 2021-02-15

**Authors:** Larissa L. Meijer, Zoë A. Schielen, Kim Y. van Ree, H. Chris Dijkerman

**Affiliations:** Experimental Psychology/Helmholtz Institute, Utrecht University, Utrecht, Netherlands

**Keywords:** itch, affective touch, C-fibers, somatosensory, relief

## Abstract

**Introduction:** Itch is a common symptom in dermatologic and other diseases and can have a severe impact on quality of life and mental health. As a proportion of patients with itch-symptoms is resistant to commonly used anti-histamine treatments, development of new treatments is desirable. Past research on pain, itch and affective touch (i.e. slow, gentle stroking of the skin activating C-tactile fibers) revealed an inhibitory relationship between affective touch and pain and between pain and itch. Given the overlap in neural processing between these three sensory submodalities, a possible interaction between affective touch and itch might be expected. This study investigated whether there is a relationship between itch and affective touch, and if so, whether affective touch inhibits itch.

**Methodology:** Itch was electrically induced with the use of electrodes that were placed at the ventral side of the wrist of 61 participants. A within-subject design was conducted with two conditions. An experimental -affective touch- condition (stroking the forearm with a soft brush at 3 cm/s) and a control -non-affective touch- condition (stroking the forearm with a soft brush at 18 cm/s). Touch was applied on the dorsal side of the forearm, the same arm as were the electrodes were placed. For each condition itch was induced for 20 min, with every 2 min a VAS-scale measurement of the level of experienced itch.

**Results:** Both types of touch reduced the experienced itch compared to baseline (*p* < 0.01, partial η^2^ = 0.67). However, affective touch had an additional significant relieving effect compared to non-affective touch (*p* = 0.03, partial η^2^= 0.08). The alleviation of itch started after 2 min of stroking and continued to increase up till 6 min, where after the relieving effect stabilized but still persisted.

**Conclusion:** This finding suggest that affective touch, as with acute pain, has a relieving effect on electrically induced itch.

## Introduction

Itch is a common symptom in dermatological diseases and is defined as “an unpleasant sensation causing the urge to scratch” (1). Itch is a commonly experienced problem, with a prevalence of 8.4% in the general population. In addition, the lifetime prevalence of chronic itch is even higher with 22%, meaning that one out of five people will experience chronic itch (2). Furthermore, the burden of itch is comparable to the burden that is experienced during chronic pain and itch symptoms can significantly impact quality of life and mental health as well (3–7). Studies indicated that higher scores on an itch intensity scale in patients with dermatological diseases causing itch were related to a higher score on depression scales (7, 8). In addition, a study by Schneider et al. (9) showed that 70% of the patients in a sample of 109 participants with dermatological diseases causing itch had one to six psychiatric disorders. Given the high prevalence and impact of itch, it seems a worldwide problem (3).

One aspect of itch that has been researched considerably is the neurophysiological basis of itch. In 1997, itch-selective neurons were discovered in humans (10). Research indicated that these neurons were part of a broader category of neurons called C-afferents. These C-afferents are characterized by their lack of myelination and therefore have a slow conducting speed (1, 10, 11). After activation of the C-fibers, the signal is transported to the dorsal horn of the spinal cord from which signals are projected to the thalamus, somatosensory cortex, sensorimotor cortex, posterior cingulate cortex (PCC), anterior cingulate cortex (ACC), insular cortex, and the basal ganglia with the putamen (12, 13). The involvement of the ACC contributes to the affective component of itch (13).

Furthermore, subsequent research implied that the itch-selective neurons were sensitive to histamine, mostly associated with acute itch experience (14, 15). However, recent research revealed that some of these neurons do not respond to histamine, but are activated by other substances and stimuli (16). Consequently, a proportion of patients with itch symptoms is resistant to commonly used anti-histamine treatments (16, 17). It seems that especially chronic itch conditions are associated with non-histamine sensitive neurons and therefore respond better to treatments targeting the nerves than the immune system (14). Given the impact of (chronic) itch, development of new suitable treatments is needed.

The development of new treatments could be inspired by research on another sensory modality that affects itch, namely pain. During the processing of pain, brain areas such as the insular cortex, cingulate cortex and premotor areas are activated (18). As mentioned, these brain areas are also highly involved in the processing of itch (13). This shows the close relationship between these senses at supraspinal level (11, 15, 18). In addition, itch and pain also seem to affect each other at a behavioral level: research confirmed that painful sensations can reduce itch sensations, which explains why we scratch our skin during itch (11, 15, 19). Scratching activates interneurons in the dorsal horn of the spinal cord. These interneurons subsequently inhibit the transduction of C-afferent signals involved in itch (20–22). Because the itch-signal is inhibited by pain, the transduction of the signal from the dorsal horn of the spinal cord toward the thalamus is reduced which results in a reduction of itch sensations.

Reducing itch by evoking pain (e.g., scratching) can only be a temporary solution as it is unpleasant and can cause serious skin inflammation when used on a permanent basis. However, the interaction between pain and other systems might provide useful information on which new interventions to reduce itch can be based. An example is the interaction between pain and affective touch. Affective touch activates another subgroup of C-afferents, known as C-tactile or CT-afferents (23–25). CT-afferents are located mainly in the hairy skin and respond to slow and gentle stroking of the skin, consisting of velocities between 1 and 10 centimeters per second, with an optimal response at 3 centimeter per second, which provides a pleasant sensation (23, 26, 27). The CT-afferents transmit signals through the dorsal horn of the spinal cord to the thalamus, insula, anterior cingulate cortex (ACC), prefrontal cortex and amygdala (28). Recent research shows that affective touch and pain influence each other as well: affective touch has an inhibitory effect on acute pain (29, 30). It seems that affective touch can inhibit pain at the level of the dorsal horn of the spinal cord by a specific inhibitory pathway related to CT-fiber input (31). In addition, Gursul et al. (32) and von Mohr et al. (33) showed that at a supraspinal level, affective touch reduces activation of areas related to pain processing, namely the insula and anterior cingulate cortex. In sum, affective touch and pain share neural characteristics that are comparable to the similarities between pain and itch. Furthermore, affective touch seems to inhibit pain, as is the case for the effect of pain on itch.

The evident similarities between brain areas involved in itch, pain and affective touch, and the inhibitory behavioral effects of pain on itch and affective touch on pain, suggest that affective touch might have a relieving effect on itch (20) and the current study will research this. Itch will be induced by an electrical current stimulator, as recent research shows that this is a reliable way of inducing itch (17, 34). The relieving effect of affective touch on itch will be evaluated by testing the decrease in perceived itch in two conditions. Affective touch will be used in the experimental condition, and non-affective touch (stroking with a velocity of 18 cm/s) will be used in the control condition. Itch induction and touch stimulation will be provided simultaneously for 20 min. This time-frame is based on recent research of Sailer et al. (35) showing that the activation of brain areas related to affective touch and the perceived pleasantness of affective touch stabilizes after 20 min. Therefore, we expect that, if there is a relieving effect of affective touch, it will persist for ~20 min. In addition, perceived pleasantness of affective touch will be monitored, and the relationship between pleasantness of affective touch and its relieving effect on itch will be researched. It is expected that experiencing affective touch as more pleasant is associated with experiencing more itch relief from affective touch (36, 37). A factor that modulates how intensely itch is experienced is the amount of attentional focus. Research suggests that a high attentional focus to bodily sensations such as itch, increases the amount of experienced itch (38). Therefore, we will additionally investigate whether there is a relation between high awareness to bodily sensations and the alleviation of itch by affective touch. We expect that people who have a high attentional focus to bodily sensations, experience less relief from affective touch (39).

## Methods and Materials

### Participants

An a priori calculation for the repeated measures ANOVA (*f* = 0.2, α err prob. = 0.05, power = 0.95, number of groups = 2, number of measurements = 11) recommend a sample size of 30. Eventually, 69 participants signed up for participation out of which 61 participants were eligible for participation. The study group consisted of 12 men (Mage = 27.50, range age = 18–28) and 49 women (Mage = 21.61, range age= 18–53). Of the participants, 54.1% were following or had finished tertiary education, 45.9% had finished secondary education. The participants were recruited through the Social and Behavioral Sciences research participation system (SONA) of Utrecht University. Participants from the age of 18 and older, and fluent in the Dutch language were eligible to participate in this experiment. People suffering from a skin condition where itch is a present symptom, like chronic itch or psoriasis, or people using a pacemaker, were excluded from this experiment. People using a pacemaker were not allowed to participate because of the electrical stimulation that could interfere with the functioning of the pacemaker. The faculty ethical review board of the University of Utrecht approved the study's protocol and all participants gave permission for participating in this experiment by means of a written informed consent.

### Materials

#### Demographical Information

To verify the inclusion and exclusion criteria the participants were asked to state their age, gender, highest completed education, whether they were suffering from skin conditions, and whether they were using a pacemaker.

#### Pain and Vigilance Attention Questionnaire (PVAQ)

To examine the awareness to bodily sensations, an adjusted version of the Pain Vigilance and Awareness Questionnaire (PVAQ) was used (40). Originally, this questionnaire was focused on pain. As claimed by van Laarhoven et al. (34), the questionnaire is suitable to investigate itch, when changing the word “pain” to “physical sensations.” This alteration had no consequences for the reliability or validity of the PVAQ. The questions of the PVAQ focus on sensing, ignoring and monitoring bodily sensations. The PVAQ consists of 16 items, which are scored on a 6-point Likert scale. Zero represents “never,” 5 represents “always.” Items 8 and 16 should be reverse-scored before the total score of the PVAQ can be calculated. A relatively low score on the PVAQ indicated low attention to bodily sensations. A relatively high score represented high attentional focus on bodily sensations.

### Itch Induction

An electrical stimulus was used to induce itch. According to Ikoma et al. (17) and van Laarhoven et al. (34), using a constant current stimulator (Isolated Bipolar Constant Current Stimulator DS7, Digitimer, United Kingdom) is a reliable way to induce itch. Nerve stimulation electrodes were attached to the ventral side of the wrist, alternately to the right or left wrist equally divided among the participants. The DS7 had a default setting where the pulse duration was set at 100 milliseconds and the compliance voltage was set at 200 volts. E-prime 2.0 (Psychology Software Tools, 2015) was used to alter the pulse duration of the DS7. A transmission of a constant stimulation of 50 Hz by having a pulse duration of 20 milliseconds was programmed. These pulses were active for 0.2 milliseconds and inactive for 19.8 milliseconds. The level of amperage (in milliampere) was individually adjusted prior to the experiment and was determined based on the participants' experienced level of itch. A stimulation period of 4 s was used to test the itch stimulation, the experienced itch was rated on the VAS. After each VAS rating, the amperage was increased, by steps of 0.1–0.2 mA, until the participants considered the experienced itch a 7 or higher on the VAS. If participants did experience itch but did not report higher than a 7 on the VAS, the highest rating of the experienced itch was registered. The corresponding amperage was used in the experiment. The level of amperage ranged from 1.80 to 4.90 (M_amperage_= 3.02, SD_amperage_= 0.76). When participants did not experience any itch or the experienced itch intensity was not rated with a three or higher, the experiment was discontinued.

### Affective and Non-Affective Touch

The (non-)affective touch stimulation was executed by stroking with a soft foundation brush. The velocity of stroking in the experimental affective touch condition was 3 centimeters per second. The velocity of stroking in the control non-affective touch condition was 18 centimeters per second. The researcher marked the length of 6 centimeters on the dorsal side of the arm to which the electrode was attached. This enabled the researcher to stroke with the correct velocity during the affective touch and non-affective touch condition (thus in the affective touch condition, the 6 cm length was stroked over 2 s, while in the non-affective touch condition the 6 cm length was stroked 3 times per second).

### Monitoring Sensations

#### Visual Analog Scale (VAS) for Itch

To measure itch, participants were asked to indicate the degree of itch they experienced on a scale of 0 to 10. Zero represented “no itch” and 10 represented “unbearable itch.”

#### VAS for Pleasantness

To measure the experienced pleasantness of stroking, participants were asked to rate the experienced pleasantness on a scale that ranged from 0 to 10, where 0 represented “very unpleasant” and 10 represented “very pleasant.” The VAS is evaluated as a reliable and valid assessment to measure itch and pleasantness (35, 41).

### Procedure

**Figure 1:**
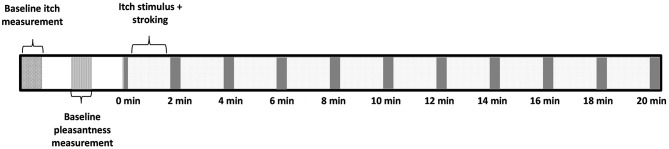
Outline in time of a single condition.

Prior to the experiment participants filled in the demographical details and the PVAQ. The baseline itch intensity was registered before each condition. Hereafter, the baseline pleasantness of touch was determined and registered by stroking the arm for 10 s over the 6 cm outline at either affective or non-affective touch velocities. The participant underwent an experimental and control condition which both had a duration of 20 min, the order was randomized between subjects. Between the conditions there was a 10 min break. Each condition had 10 blocks of 2 min simultaneous stimulation from the DS7 and stroking. Immediately following every 2 min of itch stimulation and stroking, the participants were asked to rate the experienced itch on the VAS. This took ~10 s. Hereafter the next 2 min of itch stimulation and stroking started (Figure 1).

### Statistical Analysis

Data was analyzed using SPSS Statistics (version 26). Eight participants were excluded and the experiment was discontinued because they did not experience itch. Data was checked for normal distribution. For the VAS itch intensity scores, which were analyzed with a repeated measures ANOVA, the residuals were used. According to the Shapiro–Wilk test most variables were not normally distributed, but the Shapiro–Wilk test is shown to be too sensitive in a large sample size (>50). Therefore, data was also visually inspected using the Q-Q plots and histograms, these showed that the data was approximately normally distributed. Based on these factors, together with the large sample size (>60), we decided that parametric testing was permitted (42, 43). The sphericity was mildly violated, therefore the Greenhouse-Geisser Epsilon output was used.

The VAS pleasantness scores were also checked for normality. The VAS scores for pleasantness in the affective touch condition violated the assumption of normality. The VAS scores for pleasantness in the non-affective condition did not violate the assumption of normality. The differences between the pleasantness of touch in the affective and non-affective condition were checked with a paired *t*-test.

In order to analyze the difference between the effect of affective touch and non-affective touch on itch, a repeated measures ANOVA was conducted, with touch (affective and non-affective touch) and time (baseline and the 10 timepoints within a 20-min period on which itch was measured) as independent variables and the experienced itch measured with VAS scores as dependent variable.

To analyze the changes in the relieving effect of affective touch on itch over time, a repeated measures ANOVA with contrasts (follow-up analysis) was conducted with the itch ratings from the experimental and control conditions excluding the baseline itch measurements. Every 2-min itch measurement was compared to the first 2-min itch measurement. This resulted in nine contrast analyses.

To assess the effect that attention to bodily sensations has on the effect of affective touch on itch, a Spearman correlation with the difference scores between itch ratings in the non-affective and affective touch conditions and the PVAQ scores was conducted. The mean of the 10 measurements in the non-affective condition was subtracted from that of the affective condition. Two assumptions for the Pearson correlation were violated, therefore a non-parametric Spearman correlation was conducted.

To analyze the influence of individual differences in experienced pleasantness on the relieving effect of affective touch, the difference scores of pleasantness were correlated with the difference scores of itch. The difference scores of pleasantness were calculated by subtracting the VAS pleasantness score in the non-affective condition from those in the affective condition. The difference score of itch was calculated by subtracting the mean itch VAS scores in the non-affective touch conditions from those in the affective touch condition. The assumptions of normality and linearity were violated, a Spearman correlation was conducted. All results displayed are means ± SE, unless otherwise stated. A *p* < 0.05 is considered statistically significant.

## Results

The baseline data and experimental data of itch and pleasantness and the PVAQ scores are displayed in Table 1. The baseline itch measured in the affective and non-affective condition were comparable (6.80 ± 0.16 and 6.75 ± 0.21, respectively, *t*_(60)_ = 0.23, *p* = 0.82).

**Table 1:** The mean scores, SE and range of VAS scores of the affective and non-affective touch condition, the PVAQ score and the difference scores of itch and pleasantness (*N* = 61).

	**Mean ± SE**	**Range**
**Affective touch**		
Baseline itch VAS score	6.80 ± 0.16	3.00–9.00
2-min measurement itch VAS score	3.83 ± 0.29	0.00–8.50
4-min measurement itch VAS score	3.57 ± 0.29	0.00–8.00
6-min measurement itch VAS score	3.39 ± 0.32	0.00–9.00
8-min measurement itch VAS score	3.60 ± 0.32	0.00–9.00
10-min measurement itch VAS score	3.80 ± 0.34	0.00–9.00
12-min measurement itch VAS score	3.82 ± 0.35	0.00–9.00
14-min measurement itch VAS score	4.06 ± 0.33	0.00–9.00
16-min measurement itch VAS score	4.17 ± 0.35	0.00–9.00
18-min measurement itch VAS score	4.16 ± 0.35	0.00–9.00
20-min measurement itch VAS score	4.24 ± 0.38	0.00–9.00
Baseline pleasantness VAS score	6.82 ± 0.22	0.00–10.00
**Non-Affective touch**		
Baseline itch VAS score	6.75 ± 0.21	2.00–10.00
2-min measurement itch VAS score	4.00 ± 0.34	0.00–9.00
4-min measurement itch VAS score	4.24 ± 0.34	0.00–9.00
6-min measurement itch VAS score	4.37 ± 0.34	0.00–9.00
8-min measurement itch VAS score	4.22 ± 0.35	0.00–9.00
10-min measurement itch VAS score	4.40 ± 0.33	0.00–9.00
12-min measurement itch VAS score	4.41 ± 0.36	0.00–9.00
14-min measurement itch VAS score	4.34 ± 0.35	0.00–9.00
16-min measurement itch VAS score	4.56 ± 0.36	0.00–9.00
18-min measurement itch VAS score	4.77 ± 0.37	0.00–9.50
20-min measurement itch VAS score	4.77 ± 0.38	0.00–9.50
Baseline pleasantness VAS score	5.24 ± 0.23	1.00–9.00
PVAQ Score	37.25 ± 1.35	13–62
Difference score of VAS scores for itch	0.55 ± 0.24	−3.20–5.00
Difference score of VAS scores for pleasantness	−1.58 ± 0.31	−6.00–8.50

**Figure 2:**
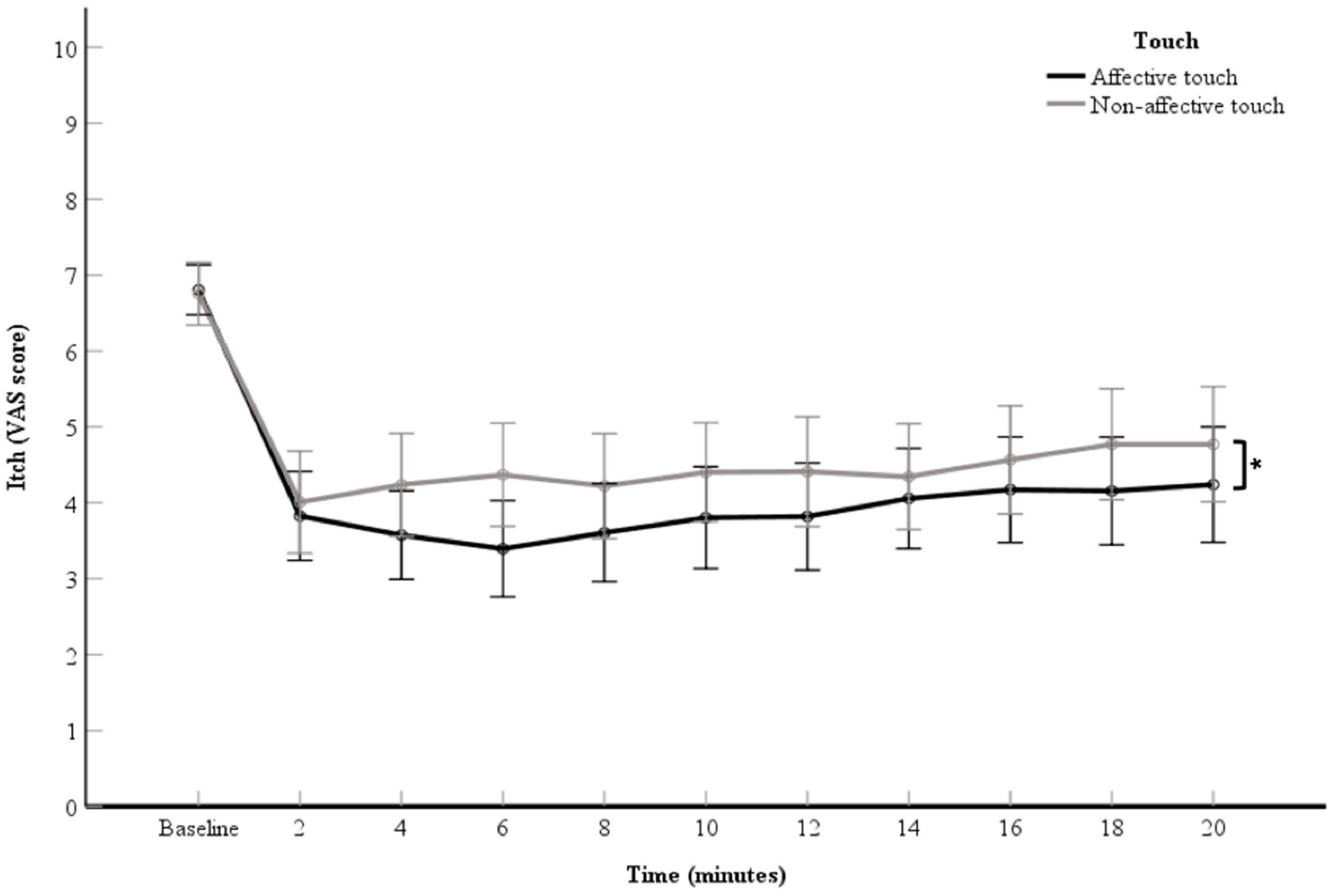
Mean of VAS scores for itch per 2-min itch measurement including the baseline measurement. The data points represent the means of the VAS scores for itch (with error bars depicting standard error). The black line represents affective touch and the gray line represents non-affective touch (*N* = 61). *p < 0.05; Displaying significant difference between type of touch.

**Figure 3:**
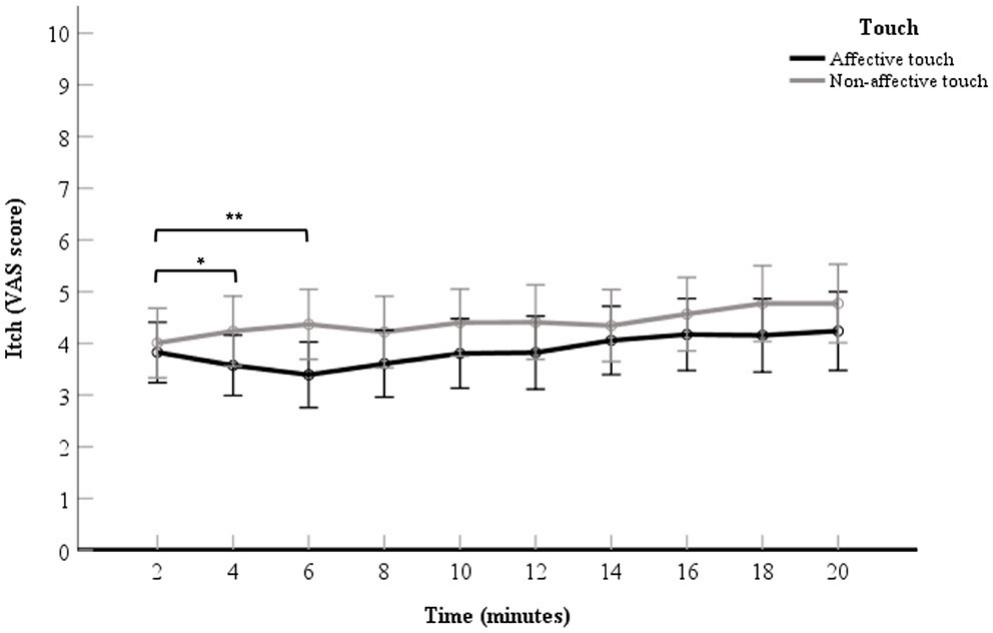
Mean of VAS scores for itch per 2-min itch measurement including the baseline measurement. The data points represent the mean of the VAS scores for itch (with error bars depicting standard error). The black line represents affective touch and the gray line represents non-affective touch (*N* = 61).

*Data of Table 1 are partly visualized in Figures 2, 3*.

A two tailed, paired samples *t-*test was used to compare the VAS scores of pleasantness for the affective touch and non-affective touch condition (Table 1). The VAS scores for pleasantness in the affective touch condition were statistically significantly higher than the VAS scores for pleasantness in the non-affective touch condition, *t*_(60)_ = 5.07, *p* < 0.01. Cohen's *d* for this test was 0.89, which can be described as large.

### Relieving Effect of Touch Relative to Baseline Itch Measurements

A 2 (touch: affective touch vs. non-affective touch) × 11 (time point: the VAS scores of the baseline itch measurement and the 10 itch measurements of after each 2-min itch stimulation) repeated measures ANOVA was used to investigate the relieving effect of touch on itch. A significant main effect for touch was obtained, *F*_(1, 60)_ = 4.87, *p* = 0.03, partial η^2^= 0.08 (Figure 2). The VAS scores for itch were significantly lower during the affective touch condition than during the non-affective touch condition (Table 1, Figure 2). A significant main effect was also reported for time point, *F*_(2.58,154.61)_ = 28.72, *p* < 0.01, partial η^2^= 0.32 (Figure 2). A contrast analysis was conducted to compare the baseline VAS scores with the other timepoints. There were significant differences between the baseline itch VAS score and the experimental itch VAS scores in both the affective and non-affective touch conditions *F*_(1, 60)_ = 121.24, *p* < 0.01, partial η^2^ = 0.67). The interaction between touch and time point was not significant, *F*_(4.92,295.14)_ = 1.75, *p* = 0.12, partial η^2^= 0.03 (Figure 2).

#### Relieving Effect of Touch for Experimental Itch Ratings

A 2 (touch: affective touch vs. non-affective touch) × 10 (time point: the VAS scores the 10 itch measurements after each 2-min itch stimulation) repeated measures ANOVA was conducted to measure the effect of touch over time. The VAS scores for itch in the affective touch condition were significantly lower than the VAS scores for itch in the non-affective touch condition, which illustrates a main effect for touch, *F*_(1, 60)_ = 5.01, *p* = 0.03, partial η^2^= 0.07. The VAS scores for itch within each condition differed significantly over time, which illustrates a main effect for time point, *F*_(2.08,124.79)_ = 3.33, *p* = 0.04.

There was no significant interaction effect found for touch × time point, *F*_(4.10,245.71)_ = 1.30, *p* = 0.27, in the ANOVA analysis. Although the interaction was not significant, as we did expect changes over time with respect to the difference in itch between conditions and after visual inspection of the data (Figure 3), it seemed of interest to conduct a follow up contrast analyses for the interaction. The 2- vs. 4-min itch measurements differed significantly, thus the difference in itch ratings between affective and non-affective touch were significantly larger at 4-min compared to 2 min, *F*_(1, 60)_ = 4.66, *p* = 0.03, partial η^2^= 0.07 (Table 2, Figure 3). A similar difference was found for the VAS scores for itch at 6-min compared to the 2-min measurement, *F*_(1, 60)_ = 9.19, *p* = < 0.01, partial η^2^= 0.13 (Table 2, Figure 3). Further details about the contrast analyses are stated in Table 2.

**Table 2:** Results of the interaction effect between touch and itch, derived from a 2 × 10 repeated measures ANOVA contrast analysis.

	**Touch** **×** **Time point**	
**Contrasts**	***F***	***p***
2 vs. 4 min	4.66	0.035^*^
2 vs. 6 min	9.19	0.004^**^
2 vs. 8 min	2.14	0.148
2 vs. 10 min	1.31	0.257
2 vs. 12 min	1.25	0.268
2 vs. 14 min	0.08	0.773
2 vs. 16 min	0.34	0.564
2 vs. 18 min	1.29	0.261
2 vs. 20 min	0.78	0.380

*For every contrast analysis, the degrees of freedom are 1.60*.

* *p < 0.05*;

** *p < 0.01*.

### Relieving Effect of Touch and Pleasantness

Spearman's correlation indicated no correlation between the difference in experienced pleasantness for affective and non-affective touch and the difference in itch ratings for the affective and non-affective touch conditions, ρ_*s*_ = −0.18, *p* = 0.17, two-tailed, *N* = 61.

### Relieving Effect of Touch and PVAQ

**Figure 4:**
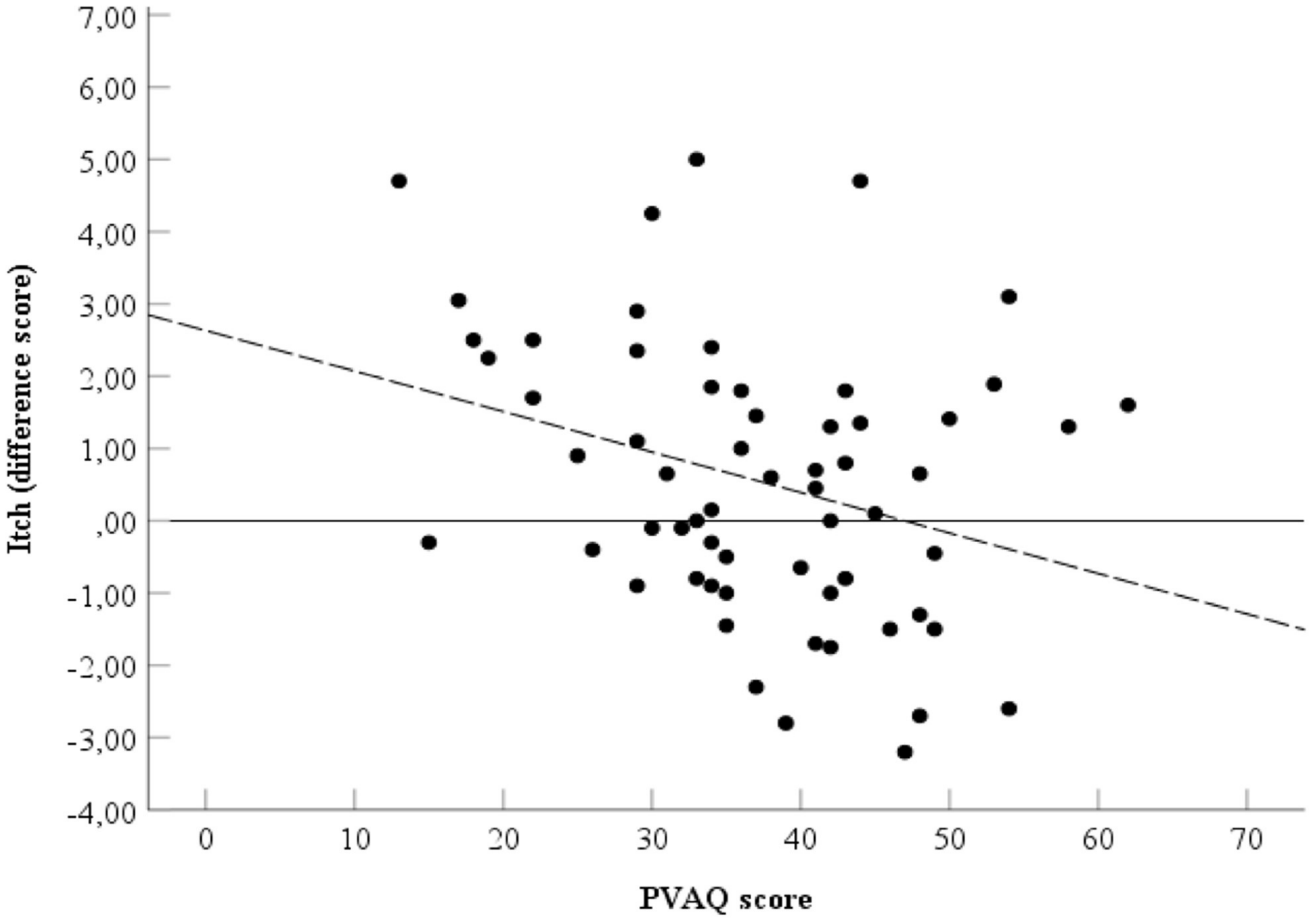
Relationship between PVAQ scores and itch. Spearman's ρ = −0.30. Black line represents point zero reference line. Dotted line represents the line of best fit.

Spearman's rho indicated the presence of a negative correlation between the PVAQ score (Table 1) and the relieving effect of affective touch, ρ_*s*_=-0.30, *p* = 0.02, two-tailed, *N* = 61 (Figure 4).

## Discussion

Past studies revealed an inhibitory relationship between affective touch and pain and between pain and itch (11, 15, 19, 30). However, as far as we know, no research has been reported on the relationship between itch and affective touch. Therefore, this study investigated whether there is a relationship between itch and affective touch and in particular, whether affective touch inhibits itch. The results showed that applying touch, either affective touch or non-affective touch, reduces itch experience. Several factors might account for this effect. First, it could be that the stroking of the arm is such a different sensation in comparison to electrically stimulated itch, that it is hard to feel both itch and stroking at the same time. Being touched might be a distraction on its own, independent of type of touch, which could explain why both types of touch reduced itch compared to baseline. On the other hand, there are studies showing that rubbing reliefs itch by activating low-threshold mechanosensitive A-fibers which inhibit itch signals in the spinal dorsal horn (44, 45). The velocity of rubbing could be compared to that of non-affective touch and might explain our findings. However, compared to rubbing, non-affective touch is applied with less force. This could influence the underlying mechanism regarding the inhibitory role on itch. Indeed, recent research in mice shows that a higher strength of stroking has a stronger inhibitory effect on itch. This implies that the strength of stroking plays an important role in itch inhibition (44). Therefore, it may be unlikely that the decline in itch experience by non-affective touch in our study is based on the underlying mechanisms of rubbing.

Interestingly, affective touch had an additional relieving effect on itch experience compared to non-affective touch. These findings suggest that affective touch alleviates itch experience more than non-affective touch. This implies that affective touch interferes with the processing of itch, resulting in a reduction of itch experience. How affective touch exactly interferes with itch is not known yet, but answers may be found in recent research into the pleasurability of scratching (46–49). As described, when we feel itchy we tend to scratch the itchy site. In addition, scratching provides a pleasant and rewarding feeling which explains its addictive property (49). Scratching activates brain regions involved in the processing of pleasantness, affection and reward e.g., insula, ACC and prefrontal regions (48–51). The involvement of this pleasant reward network in the brain could represent a top-down mechanism initiating a decrease in itch experience (49, 52). Furthermore, the pleasurability of scratching is associated with inhibition of the insula and ACC, regions which are associated with the emotional and affective evaluation of itch (49). Interestingly, these regions are also involved in the processing of affective touch (53). In addition, the pleasurability of scratching is mostly dependent on tactile sensations and it seems that on hairy parts of the body i.e., the arm or the back, CT-afferents are involved, which are also important for affective touch (47, 52). Taken together, the overlap in brain regions involved in scratching and affective touch, the involvement of CT-afferents in the pleasurability of scratching and our results show that affective touch possibly modulates itch through activation of regions involved in pleasantness and reward resulting in a decrease in itch experience. To confirm, further research should focus on the underlying process of this inhibitory relationship, for example by measuring brain activity through EEG or fMRI.

In addition, the optimal duration of applying affective touch to experience a relieving effect was investigated. Based on Sailer et al. (35), it was expected that the reduction in itch would persist for ~20 min. They stated that after 20 min, the brain areas activated by affective touch habituated to the stroking, which caused a decrease in brain activity and stabilized the experienced pleasantness of affective touch. Results of the current study showed that affective touch seems to reduce itch from 2 min after the start of appliance of affective touch and that this reduction in itch increased until 6 min after the start of stroking. After 6 min, this effect stabilized, but itch is still reduced compared to baseline. Importantly, even after 6 min, affective touch alleviated itch more than non-affective touch. The current study reported different temporal effects in comparison to the study of Sailer et al. (35), however they only researched the temporal effects of experienced pleasantness of affective touch and the accompanying brain activity. It could be that the temporal dynamics of the inhibitory effect of affective touch on itch do not depend on the perceived pleasantness of affective touch. Our results indeed show that the experienced pleasantness of affective touch does not correlate with the degree to which affective touch has a relieving effect on itch. This suggests that, even if affective touch is not experienced as pleasant, it still alleviates itch. This could be explained by CT-fiber activation and its possible independence of perceived pleasant, which is also hypothesized by Nagi et al. (54).

In addition, this research contributes to the evidence for an influence of attentional focus on the experience of bodily sensations. van Laarhoven et al. (38) stated that a high attentional focus on bodily sensations is associated with a more intense experience of itch, suggesting that attention to bodily sensation does play a role in how much affective touch will relief itch. The results of the current study showed that higher attention to bodily sensations indeed mediated the experienced itch. Being more susceptible to experiencing bodily sensations can intensify the feeling of itch or will diminish the relief from affective touch, which is in agreement with the hypothesis.

The current study was not without limitations. First, different durations of the baseline itch measurement (4 s) and the experimental itch measurements (2 min) were used, limiting the comparability of the results. We also did not assess the time course of electrical stimulated itch experience over the 20-min period without stroking. Any habituation to the electrical stimulus could therefore not be taken into account. In further research, it is recommended to add an extra control condition in which itch experience over time is measured without tactile input.

Secondly, electrical stimulation as a way to induce itch has its limitation. It has been reported that not everybody experienced the electrical stimulation as itch (55). An alternative way for future research to induce itch is using cowhage, a plant-based itch inducing substance evoking a mild itch (56).

Thirdly, 80% of the participants was female. This imbalance could have influenced the results. A recent meta-analysis into affective touch shows that females perceive affective touch as more pleasant than men (57). Furthermore, research into sex difference and itch experience shows that women report higher itch intensities compared to men. However, there was no difference in reduction of itch intensity between men and women when distracted (58). As our results show that pleasantness does not correlate with the degree of itch reduction and itch reduction itself is not influenced by gender, it may be unlikely that the skewed male/female ratio in our study influences the results. Nevertheless, the imbalance between male and female in our study should be taken into account when generalizing outcomes to the general population.

To expand fundamental knowledge on affective touch and its potential to contribute to clinical applications, future research should take individual differences into account. While some participants did not experience relief from affective touch, others reacted extremely well and experienced no itch at all after only 2 min of affective touch. These different responses could be caused by individual differences concerning tactile communication and touch perception (59). For example, Luong et al. (60) suggest that people have a stable preferred velocity of affective touch. These findings propose that individuals might respond differently to affective touch and that stroking should be adjusted to their preferred velocity.

To summarize, the current study showed that affective touch has a relieving effect on electrical stimulated itch. The relieving effect of affective touch is noticeable 2 min after the affective stroking has started, it stabilizes after 6 min, but persists up to 20 min. In addition, this effect is independent of the experienced pleasantness of affective touch. Lastly, a higher awareness of bodily sensations interferes with the relieving effect of affective touch on itch. The current study can serve as groundwork for future research in the application of affective touch as therapy for patient groups who experience itch as a significant burden. For this, it is necessary to determine how individuals with different kinds of non-histaminergic itch respond to affective touch and which characteristics result in maximal itch relief.

## Data Availability Statement

The raw data supporting the conclusions of this article will be made available by the authors, without undue reservation.

## Ethics Statement

The studies involving human participants were reviewed and approved by Faculty Ethics Review Board, Faculty of Social Sciences, Utrecht University. The participants provided their written informed consent to participate in this study.

## Author Contributions

LM: concept, supervision, writing, and editing. ZS and KR: concept, data collection, and writing. HD: concept, supervision, and writing. All authors contributed to the article and approved the submitted version.

## Conflict of Interest

The authors declare that the research was conducted in the absence of any commercial or financial relationships that could be construed as a potential conflict of interest.
